# Litter Accumulation and Nutrient Content of Roadside Plant Communities in Sichuan Basin, China

**DOI:** 10.3390/plants6030036

**Published:** 2017-08-30

**Authors:** Huiqin He, Thomas Monaco

**Affiliations:** 1 College of Resources and Environmental Engineering, Yibin University, Yibin 644000, China; huiqinhe211@sina.com; 2 US Department of Agriculture, Agricultural Research Service, Forage and Range Research Laboratory, Utah State University, Logan, UT 84322-6300, USA

**Keywords:** forest succession, plant community assembly, disturbance age, species colonization, principal component analysis

## Abstract

It is widely recognized that feedbacks exist between plant litter and plant community species composition, but this relationship is difficult to interpret over heterogeneous conditions typical of modified environments such as roadways. Given the need to expedite natural recovery of disturbed areas through restoration interventions, we characterized litter accumulation and nutrient content (i.e., organic carbon, total N, and P) and quantified their association with key plant species. Plant species cover and litter characteristics were sampled at 18 successional forest plant communities along major roadways in Sichuan Basin, western China. Variation in litter across communities was assessed with principal component analysis (PCA) and species with the highest correlation to PCA axes were determined with Pearson’s *r* coefficients. Plant communities with the longest time since road construction (i.e., 70 years) were distinctly different in litter total N and organic carbon compared to plant communities with a shorter disturbance history. We encountered 59 plant species across sampling plots, but only four rare species (i.e., frequency < 5) were strongly correlated with litter characteristics (*p* < 0.01); none of which were the most abundant where they occurred. These results highlight the importance of site-specific factors (i.e., geographic location, disturbance age) regulating plant litter across heavily disturbed landscapes and how litter characteristics and rare plant species are correlated.

## 1. Introduction

Plant litter is an important component of ecosystem functioning and nutrient cycling [1]. Litter accumulation provides ground cover and reduces soil erosion and water runoff [2]. In addition, litter creates soil microenvironments that may preferentially support seed germination of select plant species [3,4,5] and provides substrates for soil nutrient and resource pools [6,7]. Assessing litter attributes can improve our understanding of vegetation dynamics following natural disturbances as well as expedite ongoing efforts to restore plant communities [8,9]. 

While plant species and their relative contributions to bulk, mixed-species litter is widely studied, feedbacks between bulk litter and species composition are less clear. For example, when chemical composition of the litter from each species as well as their respective contribution to bulk litter accumulation are known [10], relationships between litter and species composition can be deciphered across environmental and successional gradients [11,12]. However, when litter composition and accumulation values are not available and species contributions to bulk litter are uncertain due to complex multi-species mixtures [13,14], associating litter traits and plant species composition must resort to exploratory multivariate analyses and/or correlative measures to gain further insights [15,16,17]. When considering mixed-species litter, identifying which species are correlated with litter accumulation may provide insights in plant community development across successional gradients [18,19]. For example, correlations between bulk litter traits and individual species can reveal site-specific information regarding the influence of litter on microenvironmental conditions, soil stability, and the establishment of select species within a vegetation type [3,20,21], which could be used to expedite the selection of suitable species for revegetation of disturbed areas [22,23].

Bulk litter nutrient content can also strongly influence plant community development [24,25]. Consequently, complex, mixed-species communities may contain a broad range of litter nutrient contents depending on species composition [26,27]. For example, grass and herb dominated plant communities produce relatively fine litter, characterized by high nutrient content (i.e., organic carbon, nitrogen [28]) and fast decomposition rates [29]. In contrast, coarse litter with greater lignin and woody plant structures has relatively lower nutrient content and decomposition rates due to resistance to enzymatic decomposition [30]. Correspondence between litter nutrient content and the abundance of individual species often reflects the contribution of dominant species within plant communities [31], whereas association with subordinate or rare species is largely unknown [17,32]. If rare species are habitat specialists, it is possible that they may actually show higher correlations with site-specific litter traits than dominant, generalist species [33,34].

Drastic disturbances, such as when soil and vegetation are removed and soil substrates are redistributed, often create heterogeneous vegetation patterns characterized by variable plant species composition [35] and litter characteristics [19]. Such heterogeneity within an ecosystem provides a unique setting to assess variation in litter accumulation and nutrient content [18,36]. For example, road construction unavoidably creates extensive disturbances [37,38], which results in successional plant communities where litter accumulation and nutrient content may similarly vary [39]. Consequently, in this study we collected bulk, mixed-litter samples from 54 plots located within 18 heterogeneous recovering plant communities that were previously destroyed by road construction in Sichuan Basin, China. Our objectives were to assess the variability of litter accumulation and nutrient content among plant communities and identify which species have the greatest correlation with bulk litter traits. We hypothesized that if rare species are habitat specialists, it is possible that they may actually show higher correlations with site-specific litter traits than dominant, generalist species [33,34].

## 2. Results

### 2.1. Sampling Plot Variation in Canopy Cover of Primary Life Forms

**Figure 1:**
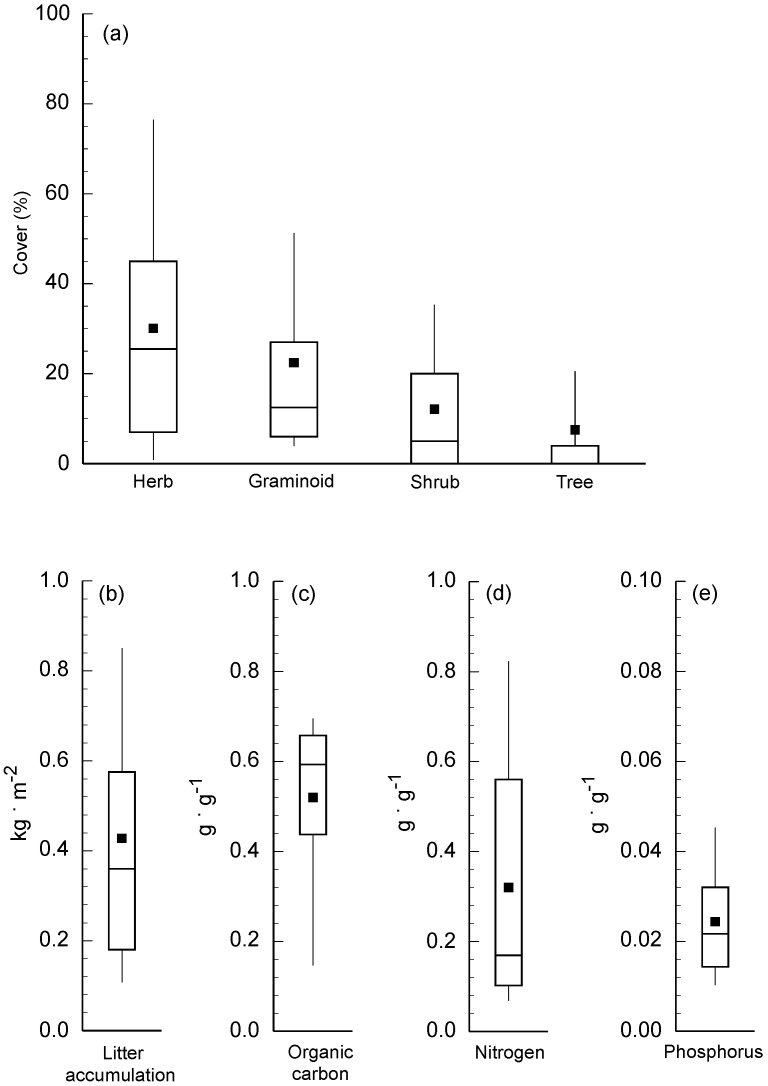
(**a**) Box-and-whisker plots showing percentage cover of plant life forms; (**b**) litter accumulation; (**c**–**e**) litter nutrient content for 54 sampling plots located along roadsides in Sichuan Basin, China. For each variable, the top, bottom, and middle lines of the box correspond to the 75th percentile, 25th percentile, and median, respectively; vertical lines extending from the bottom and top of the box correspond with the 10th and 90th percentile, respectively; and the black squares indicate the mean value. Note differences in y-axis scale.

A total of 59 species were encountered within the 54 sampling plots. Species richness was greatest for herbs (i.e., 22 species), followed by an equal number of graminoid and shrub species (15), and only 7 tree species. Cover of all life forms was variable among sampling plots (Figure 1a), illustrated by large ranges between the 25th and 75th percentiles, especially herbs. Among the 59 species, percentage cover (mean ± SE) was greatest for the large statured graminoid *Imperata cylindrica* (9.3 ± 2.7) and the herb *Artemisia argyi* (5.5 ± 1.7). Species also varied widely in the number of plots in which they occurred (i.e., frequency). Most species were actually rare based on the fact that 36 of the 59 species occurred in 5 or fewer sampling plots. By comparison, only 9 species occurred in 10 or more plots, and the two most common species (i.e., *Imperata cylindrica* and *Artemisia argyi*) also had the highest average cover. Similar to life form cover and species frequency, litter characteristics were highly variable across the sampling plots, illustrated by skewed data distributions and low correspondence between mean and median values (Figure 1b–e). 

### 2.2. Principal Component Analysis of Litter Characteristics

**Figure 2:**
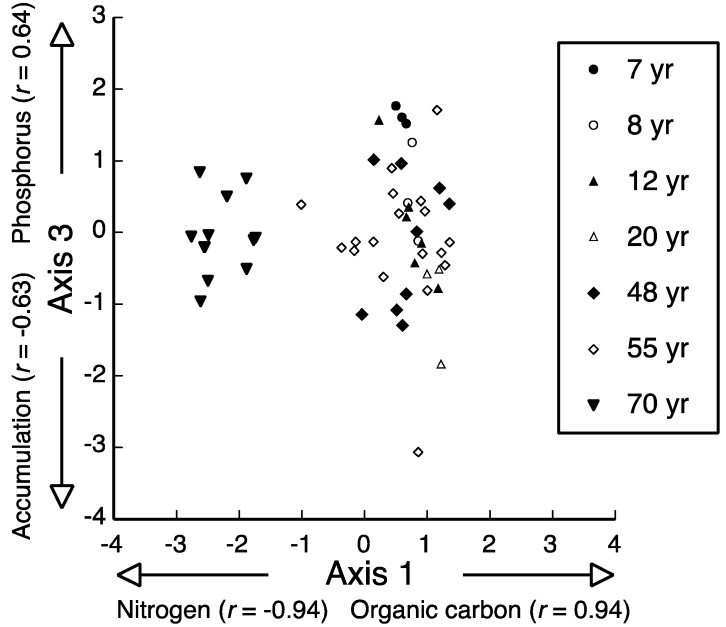
Ordination of 54 roadside sampling plots in Sichuan Basin, China, based on principal component analysis (PCA) of litter variables showing bi-plots for axes 1 and 2 (see Table 1). Plots were located adjacent to major roadways radiating from Chengdu City that varied in time since road construction (i.e., years shown with different symbols). Arrows indicate direction of Pearson’s *r* coefficient, which represents pair-wise correlation between PCA axes the litter variables.

The PCA solution explained 94.2% of total variation among the 54 plots on three axes (Table 1). Axes were best defined by the following: axis 1, inverse relationship between litter N and organic carbon; axis 2, positive relationship between litter accumulation and P; and axis 3, inverse relationship between litter accumulation and P (Table 1). Ordination of PCA scores for axes 1 and 3 showed the most distinction among sampling plots. Axis 1 emphasized that litter N was higher and organic carbon was lower for sampling plots associated the longest period since road construction (i.e., 70 years; Figure 2). In addition, axis 3 featured high variability, both within and among sampling plots, for litter accumulation and P content; however, variation was relatively lower for plots associated with the shortest period since road construction, which tended to have higher organic carbon and P than the other roads.

### 2.3. Plant Species Correlation with PCA Axes

Of the 59 species encountered, only four were significantly correlated with PCA axes (*p* < 0.01; Table 2); however, no species were correlated with axis 2 (data not shown). One tree, *Dalbergia hupeana*, and two graminoids, *Arthraxon hispidus* and *Eragrostis ferruginea*, showed negative association with PCA axis 1, and thus, greater affinity for plant communities with litter composed of higher total N, but lower organic carbon. In contrast, only one species, the shrub *Lycium chinense*, was positively correlated with axis 3, where it tended to occur in plant communities with higher P content and lower litter accumulation. Interestingly, all three of the species that were highly correlated with axis 1 only occurred in plant communities associated with the longest period since road construction (i.e., 70 years. Alternatively, *Lycium chinense* only occurred in plant communities accompanying a recovery period of 55 years. 

## 3. Discussion

The scientific literature is replete with examples illustrating a strong association between species composition and litter attributes—especially nutrient content [40,41]. Because bulk, mixed-litter content is a reflection of the plant community as a whole, dominant species often contribute a disproportionate amount to litter [42,43], which can strongly dictate nutrient cycling as well as establish plant-soil-feedbacks that favor themselves or other species in the community [44,45]. In contrast, subordinate, rare species contribute little to bulk litter accumulation but may actually be more sensitive than dominant species to site-specific litter attributes [33]. Our results support this hypothesis because only non-dominant, rare species were correlated with litter characteristics. These results may be a reflection of our study design that considered bulk, mixed litter and its relationship with heterogeneous species composition within a natural system. Indeed, experiments using single- as well as mixed-species litter in controlled settings have greater precision to evaluate how litter quantity and quality impact individual species [31,46]. Our results are also likely a consequence of rare species having greater likelihood of being correlated with litter because most species across the sampled plant communities were rare and sparsely distributed relative to the few dominant species. Nonetheless, these subordinate, rare species were correlated with litter traits and illustrate high affinity for specific litter conditions. Our results are also specific to only a few litter variables, and we recognize that other nutrients, which were not considered, might have a significant effect on species abundance across our study locations. 

The higher affinity we observed for a few subordinate species to high total litter N conditions is most apparent for one graminoid species (i.e., *Arthraxon hispidus*) and one tree species (i.e., *Dalbergia hupeana*). The graminoid is an annual grass known to flourish in ruderal environments [47,48] where soil N is often enriched [49], whereas *Dalbergia hupeana* is an important agroforestry tree that produces litter with higher N and P content relative to other co-occurring species [50]. In contrast, it is not clear why the other two species showed greater abundance on locations characterized by higher litter N content. Further experimentation is needed to understand how these site-specific conditions favor seedling establishment, growth, and persistence capacity of these rare species (i.e., [3,31,46]).

The exclusive occurrence of these four species at study locations with different periods since road construction emphasizes that site history also plays a role in species assemblages within these plant communities. Accordingly, given the site-specific nature of litter characteristics and species affinity, our results should only be viewed in the context of independent case studies for these disparate plant communities and locations. For example, longer post-disturbance periods (i.e., 55–70 years) may have contributed to unique soil development and plant community assemblages with fundamentally different litter characteristics that were particularly suitable for these rare species. Since none of the dominant species were correlated with litter patterns, it can only be speculated that the responses we observed indicate that rare species have unique requirements to initially colonize or persist over the long term in these successional forest plant communities [39]. Further research is needed to combine what is currently known about plant traits associated with colonization of roadside slopes [51] and the feedbacks between litter characteristics and the performance of individual plant species, which are typically very complex [44,46].

Greater association between litter and rare species relative to dominant species in our study stresses the importance of rare species to biodiversity of ecosystems [52]. Although most species are in fact rare in many plant communities, recent research illustrates that rare species actually contribute disproportionately to the structure and function of species assemblages [53]. In addition, rare species have been shown to disproportionately increase the range of functions provided within ecosystems, thus, their conservation value should be heightened to prevent the loss of important ecosystem services [54,55]. Roadside vegetation in the highly modified region of the Sichuan Basin thus serves as a refuge to support a high number of rare endemic species [56], where conservation is a high priority to support local plant diversity and protect numerous threatened species [57]. Our research illustrates a high degree of variation in litter characteristics among plant communities and offers a promising outlook for ongoing efforts to identify species best suited for existing roadside microhabitats (i.e., [58]).

In this study, we conclude that litter N and organic carbon explained most of the variation in litter among plant communities, and that none of the dominant species were correlated with litter characteristics. Instead, we found that four, rare and sparsely represented species were strongly associated with gradients in litter nutrient content and accumulation. High affinity of these species for specific conditions that only existed at specific locations underscores the importance of site disturbance history on litter characteristics. Consequently, our results can only offer case studies for these locations, yet they suggest that future research should pay more attention to (1) defining the contribution of both dominant and subordinate species to bulk, mixed litter pools and (2) exploring the possibility that rare species can potentially be more sensitive than dominant species to variation in litter characteristics.

## 4. Materials and Methods

### 4.1. Vegetation Sampling

**Figure 3:**
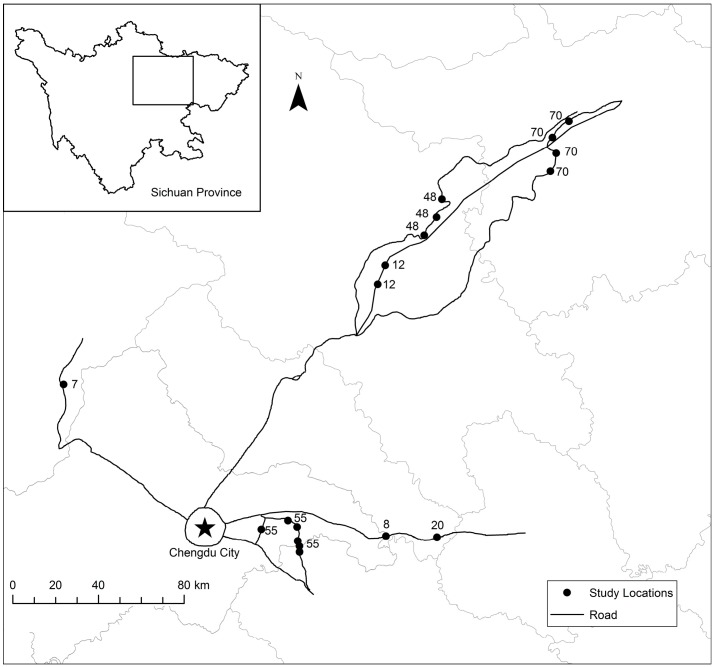
Map showing Sichuan Province in southwestern China (inset) and the 18 study locations within Sichuan Basin along major roadways near Chengdu City. Numbers indicate the period of time since road construction (i.e., years) for study locations. Gray lines represent county boundaries.

The study was conducted in Sichuan Basin, China (26°03′–34°19′ N, 97°21′–108°31′ W), a region characterized by a subtropical moist climate, with a distinct dry season between June and October. Mean annual temperature and precipitation values (1994–2014) were 17.3 °C and 826 mm, respectively [59]. The primary soil types within the basin include soils developed from either basalt or sand-shale substrates with high amounts of clay minerals, especially Fe-Al oxides [60]. The original vegetation is locally known as broad-leaved forest, often dominated by large-statured trees (i.e., *Castanopsis fargesii* and *Cyclobalanopsis glanuca* [61]). We sought to study vegetation adjacent to major roads radiating from Chengdu City where the original forest vegetation was destroyed during road construction and the current vegetation reflects various stages of natural succession over a 7–70 year period. Given the high level of urban development in this region [62], and uncertain disturbance history, large study locations to support multiple within-site sampling plots were limited. Consequently, we acquired road construction records from the Sichuan Province highway department and visited potential study locations at a set distance of 250 km along major roadways from Chengdu City. We specifically sought study locations with natural vegetation that covered an area greater than 2 hectares contained a mix of various successional stages composed of herbaceous/graminoid, shrub and tree life forms, and where no subsequent disturbances have occurred since road construction. Our search yielded 18 suitable locations that met these criteria (Figure 3). Because our objective was to assess the widest possible variation in litter and successional stages within each location and across the region, we randomly selected a 20 m × 100 m (i.e., 2000 m^2^) sampling area at each location and sampled vegetation and litter within 3, 5 m × 5 m randomly located plots, each of which were situated at least 5 m from the bottom of the slope and 10 m from other plots. This design resulted in a total of 54 sampling plots, which were subsequently analyzed as independent experimental units given the high level of within-location heterogeneity.

Vegetation was sampled in plots by visually estimating the percentage canopy cover of each species (i.e., nearest 1%). Estimates were made by one individual to avoid subjective differences among samplers. Canopy cover was defined as the proportion of ground area occupied by the aboveground parts of plants, i.e., the area covered by the vertical projection of plant canopies and tree crowns [63]. To aid in estimating cover of large trees, a cord was placed on the ground within plots to indicate canopy edges. Thus, total plot cover often exceeded 100% when canopies were multi-layered. Each species was classified by life form (i.e., herb, graminoid, shrub, and tree) and composite cover percentages were summed for each category. 

### 4.2. Litter Sampling

Within each of the 54 plots, bulk, mixed-species litter was hand-collected from 3, 1 m × 1 m subplots, sealed in paper bags, and transported to a laboratory at Sichuan University in Chengdu City. Subsamples were combined and dried in a convective oven for 48 h at 65 °C to determine litter accumulation (kg dry mass m^2^). Dried samples were thoroughly mixed and 50 g subsamples were milled through a 2 mm diameter screen. Litter organic C was analyzed according to the K_2_Cr_2_O_7_-H_2_SO_4_ oxidation method with external heating [64]. Samples were also analyzed for total nitrogen (litter N) and total phosphorus (litter P) using the Kjeldahl method and digestion in NaOH/H_2_SO_4_, respectively, using a Technicon Autoanalyzer (Seal Analytical Inc., Mequon, WI, USA). All chemical analyses were analyzed in triplicate. 

### 4.3. Statistical Analyses

Data for the 54 sampling plots were analyzed with principal component analysis (PCA) to assess variation based on the four litter characteristics. In brief, this multivariate statistical procedure identifies the relative influence of each litter characteristic on PCA axes (i.e., largest absolute eigenvector values) by placing sampling plots within an orthogonal coordinate system based on the multivariate relationship among the four litter characteristics [65]. In addition, the strength of pair-wise associations between PCA axes and percentage cover of plant species were evaluated with correlation coefficients (Pearson’s *r*) and null hypothesis tests (i.e., the true correlation coefficient is equal to zero; *α* < 0.01). All analyses were performed with Jump (JMP) version 13.0.0.

## Figures and Tables

**Table 1:** Results of principal component analysis (PCA) based on four litter variables for the 54 sampling plots located along roadsides in Sichuan Basin, China. The PCA solution identified three axes, whose eigenvalues explained 94.2% of the total variance among plots.

	Axis 1	Axis 2	Axis 3
Eigenvalues	1.8	1.2	0.8
Total variance (%)	44.1	29.7	20.4
Litter accumulation	0.028	0.775	−0.630
Litter organic carbon	0.938	−0.650	0.041
Litter total N	−0.939	0.012	0.067
Litter total P	0.065	0.764	0.642

**Table 2:** Pearson’s *r* coefficients showing correlations between species canopy cover and PCA axes 1 and 3 for 54 sampling plots located along roadways in Sichuan Basin, China. Bold text indicates significant correlations; *p* < 0.01. Frequency (i.e., number of times species was encountered) and mean percentage cover across the 54 plots are shown.

	Axis 1	Axis 3	Frequency	Cover %
**Herbs**				
*Anemone vitifolia*	0.1207	0.0985	5	2.5
*Artemisia argyi*	0.1785	0.3175	35	8.5
*Bidens pilosa*	−0.1387	0.0777	16	5.8
*Boenninghausenia albiflora*	0.0856	0.1851	3	24.7
*Cayratia japonica*	0.1271	0.1274	9	4.1
*Clematis florida*	0.0296	0.1864	3	2.7
*Commelina communis*	0.0106	0.1898	2	3.5
*Dendranthema indicum*	0.1225	0.0496	17	6.9
*Dryopteris bissetiaha*	0.0696	0.0040	10	6.9
*Epimedium brevicornum*	−0.2596	−0.0192	2	9.0
*Erigeron acer*	0.0280	0.1753	6	17.2
*Gelsemium elegans*	0.1109	0.0302	4	1.0
*Humulus japonicus*	0.1143	0.0100	7	2.7
*Iris japonica*	−0.2758	−0.0025	3	16.7
*Paederia scandens*	0.0564	0.1638	4	15.3
*Spora lygodii*	−0.2032	−0.0601	6	3.0
*Strobilanthes cusia*	−0.0082	−0.0142	6	2.7
*Taraxacum officinale*	0.1309	0.1963	4	2.5
*Torilis japonica*	0.1725	−0.035	4	3.0
*Trifolium repens*	0.1435	−0.1317	4	2.5
*Vicia carcca*	0.1194	0.2471	2	7.5
*Youngia japonica*	0.2022	0.0007	5	2.6
**Graminoids**				
*Arthraxon hispidus*	**−0.4535**	−0.0474	4	3.8
*Carex rigescens*	0.1604	−0.0622	14	10.3
*Cyperus microiria*	−0.1556	−0.2874	16	9.5
*Cynodon dactylon*	0.0269	0.0664	2	15.0
*Cymbopogon goeringii*	−0.0978	−0.074	7	18.6
*Digitaria sanguinalis*	0.0281	−0.0768	6	5.7
*Eragrostis ferruginea*	**−0.4156**	−0.0924	5	5.6
*Eriophorum vaginatum*	0.1036	0.1219	11	17.5
*Festuca arundinacea*	0.1253	−0.3227	2	26.0
*Fimbristylis dichotoma*	−0.2301	0.1107	2	9.0
*Imperata cylindrica*	0.0196	−0.2238	19	26.5
*Miscanthus sinensis*	0.1812	−0.0570	14	20.9
*Panicum brevifolium*	0.1014	0.0091	3	20.3
*Pogonatherum paniceum*	0.1480	0.0755	8	1.9
*Setaria viridis*	0.0709	0.1270	8	3.5
**Shrubs**				
*Berberis julianae*	−0.2049	−0.0133	2	8.5
*Boehmeria nivea*	0.0793	−0.0142	3	7.7
*Broussonetia papyrifera*	0.1293	−0.1376	2	9.5
*Clerodendrum bungei*	0.0958	0.1108	2	22.5
*Coriaria sinica*	−0.0082	0.0061	7	14.7
*Hedera nepalensis*	−0.0138	−0.0243	5	5.6
*Lespedeza bicolor*	−0.0929	−0.2740	6	2.3
*Lindera glauca*	0.0062	−0.1711	2	35.5
*Lycium chinense*	0.0783	**−0.4635**	2	11.0
*Pueraria lobata*	0.1002	−0.0367	3	13.3
*Rhus chinensis*	−0.3156	−0.1573	3	3.7
*Rubus corchorifolius*	−0.1512	−0.0418	6	7.2
*Solanum nigrum*	0.1061	0.2683	2	21.5
*Vitex negundo*	−0.1467	0.0362	6	16.2
*Pyracantha fortuneana*	−0.1172	0.0299	6	13.3
**Trees**				
*Cupressus funebris*	−0.2208	0.0285	4	15.5
*Dalbergia hupeana*	**−0.3564**	−0.0056	3	7.7
*Diospyros kaki*	0.0575	−0.1534	2	55.0
*Myrsine africana*	−0.0512	−0.1608	8	8.4
*Populus adenopoda*	0.0119	−0.2225	3	30.7
*Ulmus pumila*	0.0577	0.1363	2	11.5
*Vaccinium bracteatum*	−0.2469	−0.0175	2	15.0
